# Immunogenicity of Non-Living Anthrax Vaccine Candidates in Cattle and Protective Efficacy of Immune Sera in A/J Mouse Model Compared to the Sterne Live Spore Vaccine

**DOI:** 10.3390/pathogens9070557

**Published:** 2020-07-10

**Authors:** Solomon Jauro, Okechukwu C. Ndumnego, Charlotte Ellis, Angela Buys, Wolfgang Beyer, Henriette van Heerden

**Affiliations:** 1 Department of Veterinary Tropical Diseases, Faculty of Veterinary Science, University of Pretoria, Onderstepoort, Pretoria 0110, South Africa; henriette.vanheerden@up.ac.za; 2 Department of Veterinary Microbiology, Faculty of Veterinary Medicine, University of Maiduguri, Maiduguri 600230, Nigeria; 3 Moredun Scientific, Pentlands Science Park, Edinburgh EH26 0PZ, UK; o.ndumnego@moredun-scientific.com; 4 Design Biologix, Building 43b CSIR, Meiring Naude Road, Brummeria 0184, South Africa; charlotte@designbio.co.za (C.E.); angela@designbio.co.za (A.B.); 5 Department of Livestock Infectiology and Environmental Hygiene, Institute of Animal Science, University of Hohenheim, Stuttgart 70599, Germany; wolfgang.beyer@uni-hohenheim.de

**Keywords:** anthrax, non-living, vaccine, immunogenicity, cattle

## Abstract

The Sterne live spore vaccine (SLSV, *Bacillus anthracis* strain 34F2) is the veterinary vaccine of choice against anthrax though contra-indicated for use with antimicrobials. However, the use of non-living anthrax vaccine (NLAV) candidates can overcome the SLSV limitation. In this study, cattle were vaccinated with either of the NLAV (purified recombinant PA (PrPA) or crude rPA (CrPA) and formaldehyde-inactivated spores (FIS of *B. anthracis* strain 34F2) and emulsigen-D^®^/alhydrogel^®^ adjuvants) or SLSV. The immunogenicity of the NLAV and SLSV was assessed and the protective efficacies evaluated using a passive immunization mouse model. Polyclonal IgG (including the IgG1 subset) and IgM responses increased significantly across all vaccination groups after the first vaccination. Individual IgG subsets titres peaked significantly with all vaccines used after the second vaccination at week 5 and remained significant at week 12 when compared to week 0. The toxin neutralization (TNA) titres of the NLAV vaccinated cattle groups showed similar trends to those observed with the ELISA titres, except that the former were lower, but still significant, when compared to week 0. The opsonophagocytic assay indicated good antibody opsonizing responses with 75% (PrPA+FIS), 66% (CrPA+FIS) and 80% (SLSV) phagocytosis following spores opsonization. In the passive protection test, A/J mice transfused with purified IgG from cattle vaccinated with PrPA+FIS+Emulsigen-D^®^/Alhydrogel^®^ and SLSV had 73% and 75% protection from challenge with *B. anthracis* strain 34F2 spores, respectively, whereas IgG from cattle vaccinated with CrPA+FIS+Emulsigen-D^®^/Alhydrogel^®^ offered insignificant protection of 20%. There was no difference in protective immune response in cattle vaccinated twice with either the PrPA+FIS or SLSV. Moreover, PrPA+FIS did not show any residual side effects in vaccinated cattle. These results suggest that the immunogenicity and protective efficacy induced by the NLAV (PrPA+FIS) in the cattle and passive mouse protection test, respectively, are comparable to that induced by the standard SLSV.

## 1. Introduction

Anthrax is caused by the Gram-positive bacterium *Bacillus anthracis* known to primarily infect ruminants as well as other warm-blooded mammals [1]. It takes 3–5 days of incubation in the ruminant host for the disease to progress to a peracute or acute course [2]. The *B. anthracis* bacilli are responsible for systemic toxaemia and bacteraemia via its main virulence factors [3], which are encoded by two extrachromosomal plasmids, namely pXO1 and pXO2 [4]. The pXO2 encodes poly-γ-D-glutamic acid capsule (PDGA), which is poorly immunogenic and prevents *B. anthracis* phagocytosis by evading immune surveillance during the early stage of anthrax infection [5]. The aforementioned process gives the vegetative forms of the bacteria leverage to produce the tripartite toxin proteins comprising the lethal factor (LF), oedema factor (EF) and protective antigen (PA), which are encoded by the plasmid pXO1. Individually, these proteins are nontoxic until united in the binary fusion with PA as the common binding moiety, and LF and EF as the catalytic moieties. PA combines with LF and EF individually to form the binary toxins, namely lethal toxin (LT) and oedema toxin (ET), respectively. PA facilitates the translocation of LF and EF into the cells where these toxins exert deleterious effects [6,7,8]. 

Anthrax epidemics are best controlled through vaccination [9]. The Sterne live spore vaccine (SLSV) consisting of attenuated *B. anthracis* 34F2 strain (lacking the pXO2 plasmid) is used for vaccinating animals in most countries. The vaccine strain was developed by Max Sterne in the 1930s by attenuating the *B. anthracis* strain isolated from a case of bovine anthrax [10]. Since its development, SLSV has proved to be effective in protecting vaccinated animals. Booster immunization with SLSV ensures hyper immunity in goats and an early booster vaccination (within 3 months) following the first immunization has been suggested [11]. Nonetheless, SLSV is not devoid of some drawbacks, which include residual virulence in livestock and laboratory animals, adverse reaction in some animal species following vaccination and incompatibility with concurrent antibiotic treatment in disease outbreak situations [10,12,13,14,15]. The development of a vaccine that can be administered concurrently with an antibiotic in the case of disease outbreak [16], the protection of valuable wildlife or for feedlots when moving animals with unknown immune status from different locations, which require prophylactic treatment and vaccination against prevalent diseases, will be of huge benefit to the domestic/wild livestock industry. Various studies have evaluated the recombinant rPA anthrax vaccine candidate in combination with other non-living *B. anthracis* vaccine candidates in laboratory animals [3,17,18,19,20,21,22,23,24]. Recently, goats were vaccinated thrice with non-living anthrax vaccine (NLAV) candidates comprising rPA, Bacillus collagen-like protein of anthracis (BclA) and formalin inactivated *B. anthracis* 34F2 spores (FIS) (three-step vaccination schedule), and the findings showed that rPA and FIS stimulate better immune responses compared to BclA and that a two-step vaccination schedule may be sufficient [16,25]. 

In this study, rPA (crude and purified) and FIS were adjuvanted with Emulsigen-D^®^/Alhydrogel^®^. Emulsigen-D^®^ is a unique emulsion (oil-in-water) containing dimethyl-dioctadecyl ammonium bromide (DDA), which is a good stimulator of T-cell immunity and increases the antigen surface area as well as the slow release of the antigen [26,27,28]. Alhydrogel^®^ adjuvant is made up of aluminium hydroxide wet gel suspension. Alhydrogel^®^ improves the uptake of antigens by antigen-presenting cells (APCs), induces NLRP3 inflammasome complexes as well as interleukin-1 (IL-1) and interleukin-18 (IL-18) secretion and increases Th2 antibodies response [29,30,31]. In a two-step vaccination schedule, vaccine candidates were administered to cattle and the immune response and protective efficacy of the antibody-based immune responses were determined. The immune responses induced by either purified and crude rPA combined with the FIS and adjuvants were compared to the immune responses induced in SLSV-vaccinated cattle. Specific immune responses were confirmed using ELISA, in vitro toxin neutralization assay (TNA) and opsonophagocytic assay. The protective efficacy was determined using a passive mouse protection test with purified antibodies from vaccinated cattle and lethal challenge with *B. anthracis* 34F2 spores.

## 2. Results

### 2.1. Humoral Immune Response of Living and Non-Living Anthrax Vaccines in Cattle

The immunogenicity of the non-living vaccines was compared with SLSV. Sera from the animals were tested for IgG and IgM against PA and FIS, lethal toxin neutralization as well as for immunoglobin subclasses, IgG1 and IgG2. The opsonophagocytosis of induced antibodies were also investigated.

**Figure 1:**
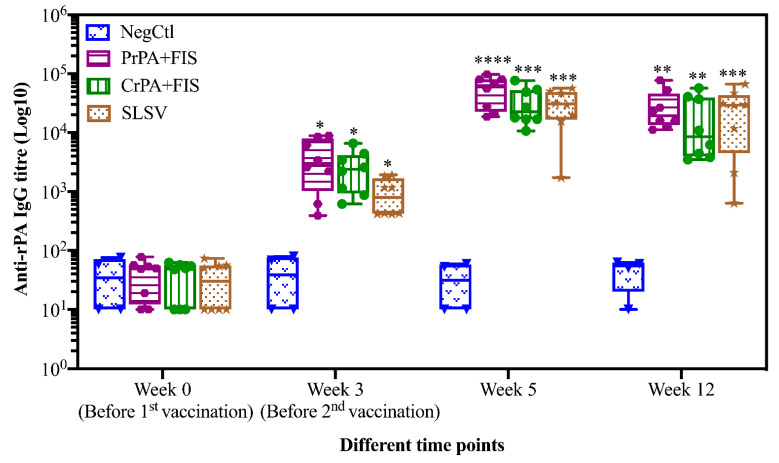
Anti-recombinant protective antigen (rPA) IgG-titres in vaccinated cattle presented as box and whisker plots. The cattle were vaccinated twice at week 0 and 3 with PrPA+FIS+Emulsigen-D^®^/Alhydrogel^®^ adjuvants (n = 8), CrPA+FIS+Emulsigen-D^®^/Alhydrogel^®^ adjuvants (n = 8), Sterne live spore vaccine (SLSV) (n = 8) and NegCtl (Emulsigen-D^®^/Alhydrogel^®^ adjuvants) (n = 4) with sera collected before the vaccinations at week 0 and 3 as well as samples collected at week 5 and 12. Sera dilution started at a concentration of 1:100 and values <50 were given an arbitrary value of 10. IgG titres in each vaccinated group were compared to the respective pre-immune titres. The significant values between groups are presented as **** *p* < 0.0001, *** *p* < 0.001, ** *p* < 0.01 and * *p* ≤ 0.05. PrPA: Purified recombinant protective antigen, CrPA: Crude recombinant protective antigen FIS: Formalin inactivated spores, SLSV: Sterne live spore vaccine, NegCtl: Negative control vaccinated with Emulsigen-D^®^/Alhydrogel^®^ adjuvants.

**Figure 2:**
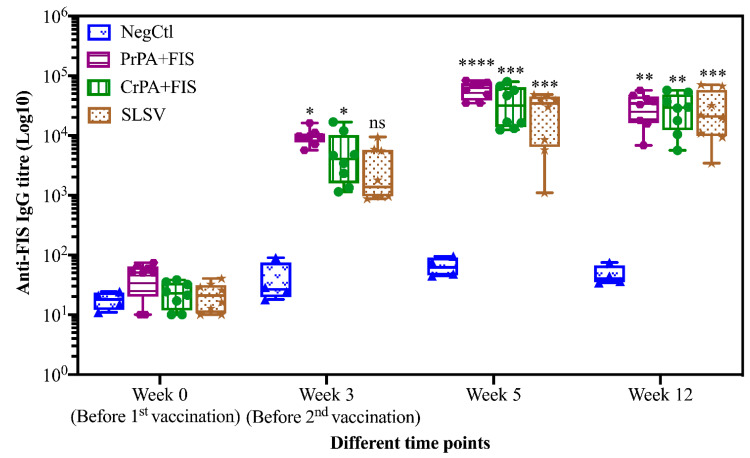
Anti-FIS IgG-titres in vaccinated cattle presented as box and whisker plots. The cattle were vaccinated twice at week 0 and 3 with PrPA+FIS+Emulsigen-D^®^/Alhydrogel^®^ adjuvants (n = 8), CrPA+FIS+Emulsigen-D^®^/Alhydrogel^®^ adjuvants (n = 8), SLSV (n = 8) and NegCtl (Emulsigen-D^®^/Alhydrogel^®^ adjuvants) (n = 4) with sera collected before the vaccinations at week 0 and 3 as well as samples collected at week 5 and 12. Sera dilution started at a concentration of 1:100 and values <50 were given an arbitrary value of 10. IgG titres in each vaccinated group were compared to the pre-immune titres. The significant values between groups are presented as **** *p* < 0.0001, *** *p* < 0.001, ** *p* < 0.01, * *p* ≤ 0.05. PrPA: Purified recombinant protective antigen, CrPA: Crude recombinant protective antigen FIS: Formalin inactivated spores, SLSV: Sterne live spore vaccine, NegCtl: Negative control vaccinated with Emulsigen-D^®^/Alhydrogel^®^ adjuvants.

Mean IgG titres against rPA rose significantly at week 3 for CrPA+FIS+Emulsigen-D^®^/Alhydrogel^®^, PrPA+FIS+Emulsigen-D^®^/Alhydrogel^®^ and SLSV. The anti-rPA IgG titres were significantly higher for CrPA+FIS+Emulsigen-D^®^/Alhydrogel^®^ and PrPA+FIS+Emulsigen-D^®^/Alhydrogel^®^ at week 5 (two weeks after the second vaccination) and week 12 when compared to the titres before vaccination (Figure 1). Similarly, the mean IgG titres against FIS increased significantly from week 3 for CrPA+FIS+Emulsigen-D^®^/Alhydrogel^®^ and PrPA+FIS+Emulsigen-D^®^/Alhydrogel^®^ but the anti-FIS IgG titres’ increase were insignificant for SLSV. At week 5 (two weeks after the second vaccination), the mean IgG titres against FIS increased was highly significantly for CrPA+FIS+Emulsigen-D^®^/Alhydrogel^®^ and PrPA+FIS+Emulsigen-D^®^/Alhydrogel^®^ and remained significant at week 12 (Figure 2). 

### 2.2. Immunoglobulins IgM and IgG Subclasses Titres

**Figure 3:**
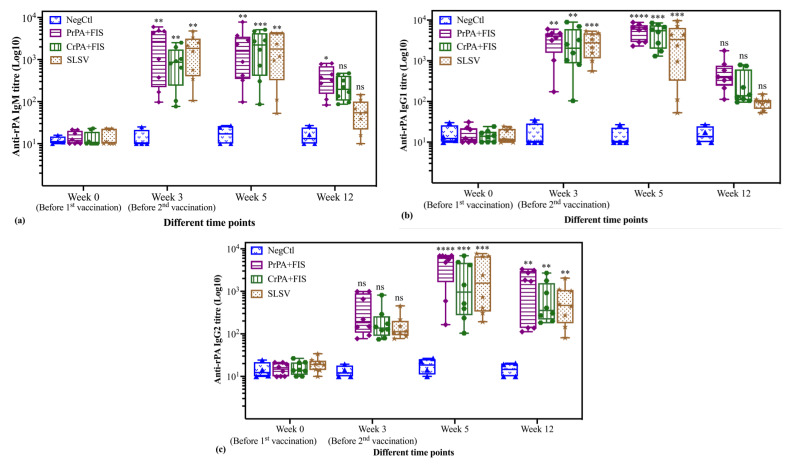
The anti-rPA IgM and IgG subclasses (IgG1 and IgG2) ELISA titres of cattle are vaccinated at week 0 and 3 with either PrPA+FIS+Emulsigen-D^®^/Alhydrogel^®^ adjuvants (n = 8), CrPA+FIS+Emulsigen-D^®^/Alhydrogel^®^ adjuvants (n = 8), SLSV (n = 8) and Emulsigen-D^®^/Alhydrogel^®^ adjuvants (NegCtl) (n = 4) are presented as box and whisker plots. The cattle sera samples were collected before the vaccinations at week 0 and 3 as well as at week 5 and 12. Sera dilution started at a concentration of 1:50 and values below the (<50) were given an arbitrary value of 10. (**a**): Anti-rPA IgM ELISA titres, (**b**): Anti-rPA IgG1 ELISA titres and (**c**): Anti-rPA IgG2 ELISA titres. The significant values were presented as **** *p* < 0.0001, *** *p* < 0.001, ** *p* < 0.01, * *p* ≤ 0.05, ns = not significant. PrPA: Purified recombinant protective antigen, CrPA: Crude recombinant protective antigen FIS: Formalin inactivated spores, SLSV: Sterne live spore vaccine, NegCtl: Negative control vaccinated with Emulsigen-D^®^/Alhydrogel^®^ adjuvants.

The antibody subclasses’ responses against rPA in vaccinated cattle groups after the first and second vaccination are presented in Figure 3a–c. The anti-rPA IgM titres exhibited significant elevation against PrPA+FIS+Emulsigen-D^®^/Alhydrogel^®^, CrPA+FIS+Emulsigen-D^®^/Alhydrogel^®^ and SLSV after the first and second vaccination, before decreasing at week 12. The IgM titres at week 12 were still significant for PrPA+FIS+Emulsigen-D^®^/Alhydrogel^®^ but not significantly higher for the +FIS+Emulsigen-D^®^/Alhydrogel^®^ and SLSV group when compared to week 0. Thus, the group vaccinated with PrPA+FIS+Emulsigen-D^®^/Alhydrogel^®^ still had significantly higher titres, despite the decline in the IgM levels (Figure 3a). The anti-rPA IgG1 titres also showed a significant increase for PrPA+FIS+Emulsigen-D^®^/Alhydrogel^®^, CrPA+FIS+Emulsigen-D^®^/Alhydrogel^®^, and SLSV at week 3 and, at week 5, the anti-rPA IgG1 titres for PrPA+FIS+Emulsigen-D^®^/Alhydrogel^®^, CrPA+FIS+Emulsigen-D^®^/Alhydrogel^®^, and SLSV increase were highly significant. However, the anti-rPA IgG1 titres declined below significance at week 12 for PrPA+FIS+Emulsigen-D^®^/Alhydrogel^®^, CrPA+FIS+Emulsigen-D^®^/Alhydrogel^®^ and SLSV (Figure 3b). The anti-rPA IgG2 titres at week 3 were not different from the pre-vaccination titres for all the vaccine groups. However, analysis at week 5 and 12 showed highly significant increases for anti-rPA IgG2 titres in all vaccine (Figure 3c). 

**Figure 4:**
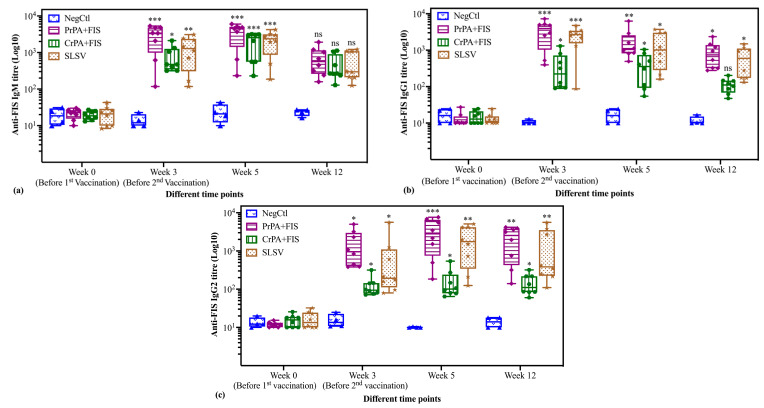
The anti-FIS IgM and IgG subclasses (IgG1 and IgG2) ELISA titres of cattle are vaccinated at week 0 and 3 with either PrPA+FIS+Emulsigen-D^®^/Alhydrogel^®^ adjuvants (n = 8), CrPA+FIS+Emulsigen-D^®^/Alhydrogel^®^ adjuvants (n = 8), SLSV (n = 8) and Emulsigen-D^®^/Alhydrogel^®^ adjuvants (NegCtl) (n = 4) are presented as box and whisker plots. The cattle sera samples were collected before the vaccinations at week 0 and 3 as well as at week 5 and 12. Sera dilution started at a concentration of 1:50 and values below the (<50) were given an arbitrary value of 10. (**a**): Anti-FIS IgM ELISA titres, (**b**): Anti-FIS IgG1 ELISA titres and (**c**): Anti-FIS IgG2 ELISA titres. The significant values were presented as **** *p* < 0.0001, *** *p* < 0.001, ** *p* < 0.01, * *p* ≤ 0.05, ns = not significant. PrPA: Purified recombinant protective antigen, CrPA: Crude recombinant protective antigen FIS: Formalin inactivated spores, SLSV: Sterne live spore vaccine, NegCtl: Negative control vaccinated with Emulsigen-D^®^/Alhydrogel^®^ adjuvants.

The anti-FIS IgM and IgG subclasses response in vaccinated cattle groups after the first and the second vaccination are shown in Figure 4a–c. The anti-FIS IgM titres after the first vaccination showed a significant increase for PrPA+FIS+Emulsigen-D^®^/Alhydrogel^®^, CrPA+FIS+Emulsigen-D^®^/Alhydrogel^®^, and SLSV. The increase in IgM titres at week 5 after the second vaccination for PrPA+FIS+Emulsigen-D^®^/Alhydrogel^®^, CrPA+FIS+Emulsigen-D^®^/Alhydrogel^®^ and SLSV were highly significant, however these were insignificant at week 12 across all the vaccine groups when compared to week 0 (Figure 4a). The mean anti-FIS IgG1 titres also showed a significant increase for PrPA+FIS+Emulsigen-D^®^/Alhydrogel^®^, SLSV and CrPA+FIS+Emulsigen-D^®^/Alhydrogel^®^ at week 3 and retained significance at week 5 across all vaccine groups. The anti-FIS IgG1 mean titres level decreased but were still significant at week 12 for SLSV and PrPA+FIS+Emulsigen-D^®^/Alhydrogel^®^ but not significant for CrPA+FIS+Emulsigen-D^®^/Alhydrogel^®^ (Figure 4b). The anti-FIS IgG2 mean titres’ increases were significant for PrPA+FIS+Emulsigen-D^®^/Alhydrogel^®^, CrPA+FIS+Emulsigen-D^®^/Alhydrogel^®^ and SLSV at week 3, increased further at week 5 for PrPA+FIS+Emulsigen-D^®^/Alhydrogel^®^ and SLSV, while declining (but still significantly higher than pre-vaccination titres) for CrPA+FIS+Emulsigen-D^®^/Alhydrogel^®^. At week 12, the anti-FIS IgG2 mean titres were significant for PrPA+FIS+Emulsigen-D^®^/Alhydrogel^®^, CrPA+FIS+Emulsigen-D^®^/Alhydrogel^®^ and SLSV (Figure 4c). The immunoglobulins subclasses mean titres against rPA and FIS of NegCtl group consisting of cattle vaccinated with adjuvants did not change significantly across all the timepoints when compared to the titres at week 0. 

**Figure 5:**
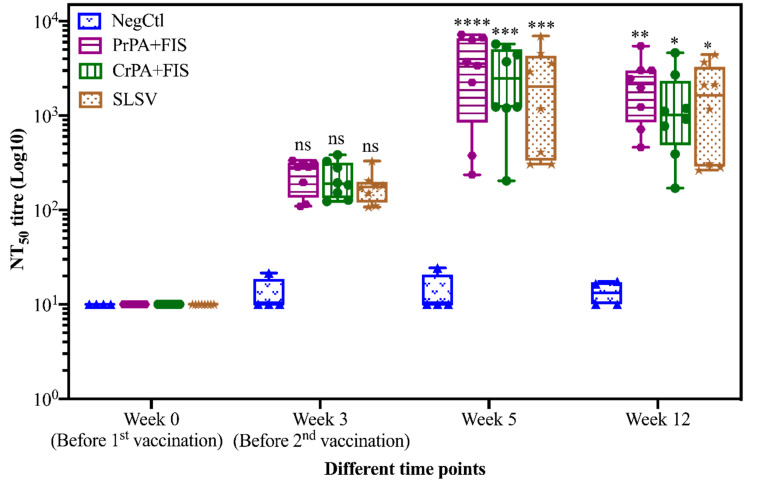
Lethal toxin neutralizing titres in vaccinated cattle. The cattle were vaccinated twice at week 0 and 3 with PrPA+FIS+Emulsigen-D^®^/Alhydrogel^®^ adjuvants (n = 8), CrPA+FIS+Emulsigen-D^®^/Alhydrogel^®^ adjuvants (n = 8), SLSV (n = 8) and NegCtl (Emulsigen-D^®^/Alhydrogel^®^ adjuvants) (n = 4) with sera collected before the vaccinations at week 0 and 3 as well as at week 5 and 12. Sera with no detectable toxin-neutralizing titres were given an arbitrary value of 10 and sera dilution started at a concentration of 1:50. Neutralizing titres in each vaccinated group were compared to the respective pre-immune titres. The significant values between groups are presented as **** *p* < 0.0001, *** *p* < 0.001, ** *p* < 0.01, * *p* ≤ 0.0.5, ns = not significant. PrPA: Purified recombinant protective antigen, CrPA: Crude recombinant protective antigen FIS: Formalin inactivated spores, SLSV: Sterne live spore vaccine, NegCtl: Negative control vaccinated with Emulsigen-D^®^/Alhydrogel^®^ adjuvants.

The lethal toxin neutralizing antibodies (NT_50_) titres of cattle vaccinated with NLAV, SLSV and adjuvant (NegCtl) are shown in Figure 5. The lethal toxin neutralizing antibody titres increased (though not significantly) across all vaccination groups after the first vaccination at week 3. The NT_50_ titres for all vaccine groups increased significantly at week 5 and 12 when compared to pre-vaccination titres. (Figure 5). The NT_50_ remained below the detection limit among the NegCtl throughout the period of study.

### 2.3. Opsonising Ability of Vaccine-Induced Antibodies 

**Figure 6:**
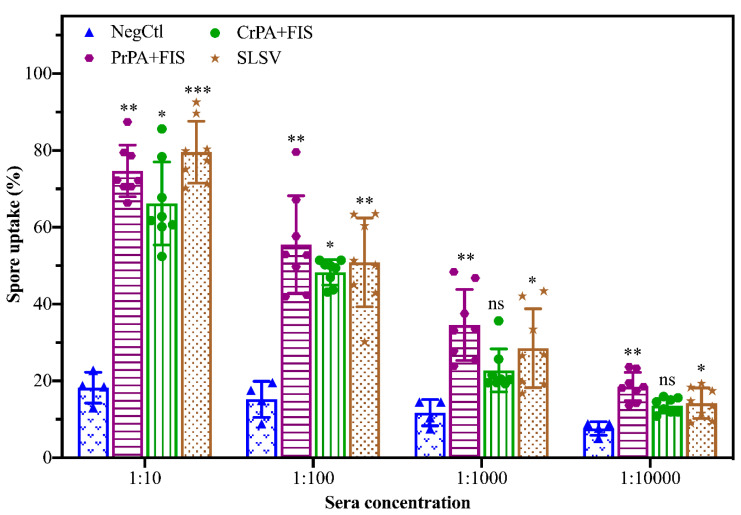
*Bacillus anthracis* 34F2 spores’ uptake (phagocytosis) by RAW 267.7 macrophages following incubation with varying dilutions of sera from vaccinated and negative control groups. The mean value of spore uptake is presented in the form of bar charts with the three standard deviations. The level of opsonophagocytosis of each group at every dilution was compared to the negative control (NegCtl) of each dilution. The significant values were presented as, *** *p* < 0.001, ** *p* < 0.01, * *p* ≤ 0.05, ns = not significant. PrPA: Purified recombinant protective antigen, CrPA: Crude recombinant protective antigen FIS: Formalin inactivated spores, SLSV: Sterne live spore vaccine, NegCtl: Negative control vaccinated with Emulsigen-D^®^/Alhydrogel^®^ adjuvants.

Sera collected at week 5 from cattle vaccinated with NLAV and SLSV were used to determine the potential of induced antibodies to opsonize *B. anthracis* spores, thereby enabling spores’ phagocytosis by RAW 264.7 macrophage (Figure 6). The macrophages showed a high level of spore uptake at 1:10 sera dilution for both SLSV and NLAV vaccine groups. The RAW 264.7 macrophages showed 80%, 75% and 66% spore uptake following treatment with immune sera from SLSV, PrPA+FIS+Emulsigen-D^®^/Alhydrogel^®^ and CrPA+FIS+Emulsigen-D^®^/Alhydrogel^®^ vaccine groups, respectively. The NegCtl showed the least level of macrophage spore uptake (17%) following incubation with sera (Figure 6). The spore uptake at 1:100, 1:1000 and 1:10000 dilutions of sera from the SLSV and PrPA+FIS+Emulsigen-D^®^/Alhydrogel^®^ vaccine groups in the opsonophagocytosis assay remained significantly high when compared to the negative control sera (Figure 6). Sera from CrPA+FIS+Emulsigen-D^®^/Alhydrogel^®^ did not show a significant macrophage spore uptake at dilutions above 1:100. 

### 2.4. Protection Conferred on A/J Mice by Antibodies from Cattle Immune Sera 

**Figure 7:**
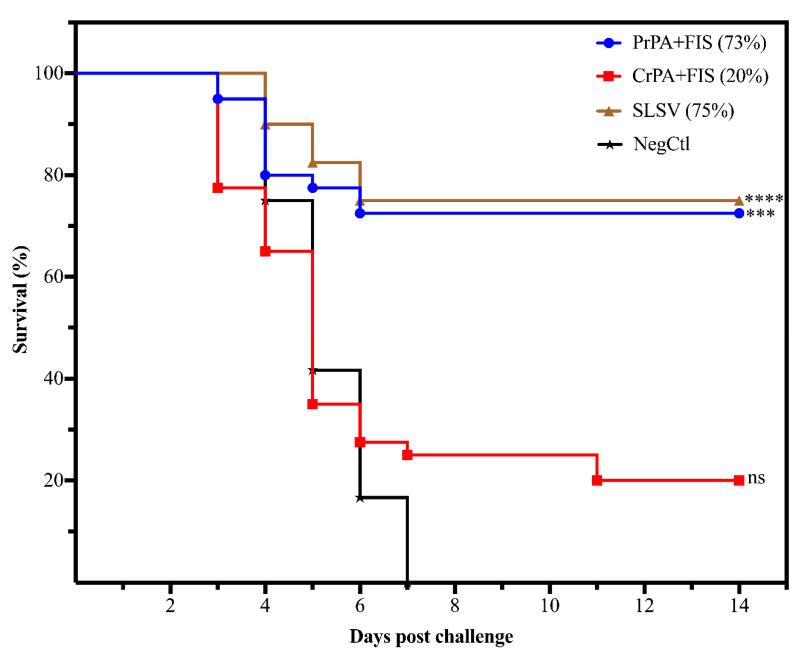
The cumulative mice survival curve following passive in vivo transfer of polyclonal IgG purified from cattle immune sera A/J mice. The mice were lethally challenged with 2.16 × 10^5^ *B. anthracis* 34F2 spores. The sera are from cattle vaccinated twice (week 0 and 3) with either PrPA+FIS, CrPA+FIS, SLSV groups adjuvanted with emulsigen-D^®^/alhydrogel^®^ and a NegCtl group (also see Table 2 for the number of mice allocated to each group). The survival rate in the Log-rank (Mantel–Cox) test was compared to the NegCtl group. The significant values between groups are presented as **** *p* < 0.0001, *** *p* < 0.001, ns = not significant. PrPA: Purified recombinant protective antigen, CrPA: Crude recombinant protective antigen, FIS: Formalin inactivated spores, NegCtl: Negative control vaccinated with Emulsigen-D^®^/Alhydrogel^®^ adjuvants.

The ability of the polyclonal IgG purified from cattle vaccinated with NLAV and SLSV to protect A/J mice from challenge with toxigenic *B. anthracis* 34F2 strain spores was determined using a passive mouse protection model. The NegCtl group consisting of mice with antibodies from cattle vaccinated, with the adjuvants only died 3–7 days following the challenge (Figure 7). A significant level of protection was seen among groups of A/J mice that were transfused with antibodies from PrPA+FIS+Emulsigen-D^®^/Alhydrogel^®^ and SLSV-vaccinated cattle recorded 73% and 75% A/J mice protection. However, IgG from CrPA+FIS+Emulsigen-D^®^/Alhydrogel^®^ vaccinated cattle were unable to confer significant protection to the A/J mice, with only 20% of the A/J mice protected from the lethal effect of *B. anthracis* 34F2 strain spores. 

Furthermore, we evaluated the relationship between the humoral and neutralizing antibody titres from the vaccine groups and the level of protection conferred to A/J mice following lethal challenge. Our findings revealed there is a correlation between anti-rPA, anti-FIS and NT_50_ antibody titres from the group vaccinated with PrPA+FIS+Emulsigen-D^®^/Alhydrogel^®^ and SLSV and the rate of survival in passively challenged A/J mice (Table 1). However, there was no correlation between the survival rate of the passively challenged A/J mice and the anti-rPA, anti-FIS and NT_50_ antibody titres from the CrPA+FIS+Emulsigen-D^®^/Alhydrogel^®^ vaccinated group. 

## 3. Discussion

SLSV, as a live spore vaccine, is effective in the control of anthrax in livestock globally and has been the vaccine of choice in veterinary practice since the initial largescale production immunization trials in the 1940s [10,32]. Despite the success achieved with the SLSV, several drawbacks, such as residual virulence in some vaccinated animals and contraindications in anthrax outbreak situations due to its incompatibility with antibiotics, remain [13,14,33]. NLAV can be used in these scenarios simultaneously with antibiotics without interference on the immunogenicity of the vaccine. Various components of *B. anthracis,* such as PA, BclA, BxpB, LF and EF as well as the inactivated form of the whole *B. anthracis* spore, have been exhaustively studied alone or in combination for immunogenicity [3,12,16,17,25,34,35,36,37,38,39,40]. Among them, PA stands out as the major immunogenic component with the potential of stimulating toxin-neutralizing antibodies [41,42]. Other studies have reported an increase in immunogenicity and protection when PA is used in combination with other immunogenic components of *B. anthracis* or FIS [16,17,19,20,23,25,39,40,43]. Most of these studies were conducted in laboratory animals except the studies by [25] and [16] were conducted in goats. These studies [16,25] reported that combination of rPA and FIS provided a significant immune response in the three-step vaccination schedule in goats, as well as hypothesizing that a two-step vaccination schedule might provide sufficient protection based on the immune response observed in the goats. Therefore, in our study, cattle were vaccinated twice with NLAV (PrPA+FIS and CrPA+FIS) with Emulsigen-D^®^/Alhydrogel^®^ and compared to the SLSV. The PrPA+FIS+Emulsigen-D^®^/Alhydrogel^®^ and CrPA+FIS+Emulsigen-D^®^/Alhydrogel^®^ and SLSV vaccinated groups provided 73%, 20% and 75% protection, respectively, to mice that were passively immunized with purified cattle antibodies and challenged with toxigenic spores of *B. anthracis*. The negative control mice from cattle sera vaccinated twice with adjuvants only died within 7 days. Our results confirmed that a two-step vaccination schedule of PrPA+FIS+Emulsigen-D^®^/Alhydrogel^®^ in cattle provide the same level of protection in the mouse protection assay as a two-step vaccination with SLSV. Ndumnego et al. [11] showed that two-step vaccine schedule (three months apart) of SLSV provided maximum protection to goats and also that the thrice vaccinated NLAV (consisting of a rPA+rBclA+FIS+lipopeptide adjuvant) can probably be reduced to a two-step vaccination schedule based on the immune response in goats [11,16]. A Pam_3_Cys-SK_4_ lipopeptide adjuvant known to enhance humoral immune response was used in these studies [44]. However, this was replaced in our study by the emulsigen-D^®^/alhydrogel^®^ adjuvants licensed to our industrial partner Design Biologix with similar results. 

The immunoglobulin subclasses titres exhibited a balance between TH1 and TH2-type responses. Even though the TH2-type immune response dominates the response at week 5, as seen with IgG2 in both NLAV and SLSV against FIS, a similar trend is also seen with IgG1 and IgG2 response against rPA at the same timepoint, which signifies the stimulation of TH1-type response as well. The IgG1 titres declined, but were still significant at week 12, unlike IgG2, which maintained the trends of titres from week 5 at week 12. However, the dynamics of the immune response switch between the immunoglobulin subclasses cannot be fully elucidated, as only four timepoints only are reported in the 12 weeks of this study. 

Solely PA-based vaccines were reported to be less protective against virulent challenge compared to the live spore vaccine [17,20,45]. The combination of PA and FIS in vaccine formulation conferred better protection [17]. Hence, the presence of FIS in our formulation may be associated with the level of protection in the passive mouse protection test. The anti-PA, anti-FIS IgG and TNA titres of cattle vaccinated with the purified rPA+FIS+Emulsigen-D^®^/Alhydrogel^®^ correlated to the rate of protection achieved in the passive mouse protection test (Table 1). This is similar to the report by Ndumnego et al. [16], with a positive correlation between anti-PA, anti-FIS and TNA titres of vaccinated goats and protection observed in passive mouse protection test. 

Interestingly, the rate of protection recorded in the cattle group vaccinated with CrPA+FIS+Emulsigen-D^®^/Alhydrogel^®^ did not correlate with the antibody titres obtained against rPA and FIS as well as to the NT_50_. Obviously, the purity of the rPA used in the PrPA+FIS+Emulsigen-D^®^/Alhydrogel^®^ vaccine was of importance for the rate of survival recorded in the mouse trial. The CrPA+FIS+Emulsigen-D^®^/Alhydrogel^®^ vaccine was included in this study as a lower-cost vaccine prototype, as opposed to the more expensive PrPA+FIS+Emulsigen-D^®^/Alhydrogel^®^ vaccine, which necessitates the purification of rPA by affinity chromatography. The low survival rate of mice after passive immunization with sera of CrPA+FIS+Emulsigen-D^®^/Alhydrogel^®^ vaccinated cattle could be due to restricted access to B-cell epitopes in the crude rPA preparation. This may result in the blockage of the PA epitopes that are responsible for the inducement of protective antibodies, as previously reported by Crowe et al. [46]. Additionally, Crowe et al. [46] observed that peptide-specific antibodies against the furin cleavage, ligand-binding and receptor binding regions of PA are responsible for neutralization (in vitro) with the furin cleavage site mediating the best protection in vitro while displaying less protection in vivo. Antibodies against the receptor-binding site showed the most robust protection in vivo, despite displaying lower protection in vitro. Further work will be needed to confirm this. Moreover, the presence of the array of proteins in the CrPA+FIS+Emulsigen-D^®^/Alhydrogel^®^ formulation may have stimulated non-specific antibody responses, adding to the overall titres measured in the whole-cell FIS ELISA [47]. In our study, we revealed that polyclonal antibodies from cattle vaccinated with PrPA+FIS+Emulsigen-D^®^/Alhydrogel^®^, CrPA+FIS+Emulsigen-D^®^/Alhydrogel^®^ and SLSV were able to opsonize and enable the uptake of 75%, 66% and 80% spores by macrophages. The opsonization of spores may substantially contribute to the rate of killing of phagocytosed spores by macrophages [37].

## 4. Materials and Methods

### 4.1. Recombinant Protein Expression and Purification

The construction of PA_83_ coupled to the His-tag expressing pStaby1.2 plasmid in *Escherichia coli* SE1 was done by Delphi Genetic, Belgium, using the PA83 primers and method, as described by Hahn et al (2004), and was designated pStaby1.2-PA83.1. The pStaby1.2-PA83.1 transformed into *E. coli* SE1 was expressed by culturing in Luria–Bertani (LB) broth medium at 25 °C in a shaking incubator and observed until an optical density of 0.8 at 600 nm was reached. Then, protein expression was induced with 0.3 mM of isopropyl-beta-D-thiogalactopyranoside (IPTG) and the *E. coli* SE1 were further incubated overnight at 25 °C before harvesting by centrifuging at 2500 xg for 35 min. The pellet was washed with double distilled water. The protein was purified following the lysis of *E. coli* SE1 by suspending the pellet of the bacteria in a lysis buffer (400 mmol/l NaCl, 50 mmol/l NaH_2_PO_4_ and 20 mmol/l Tris; pH 7.85), followed by three freeze–thawing cycles of −20 and 4 °C, respectively, in each cycle. The lysing process also included two cycles of sonication immediately after the freezing and thawing cycles. Each sonication cycle consisted of 20 s of sonication and 10 s without sonication on ice using a probe sonicator (BioLogics, Manassas, VA, USA) three times. Following sonification, the lysate was centrifuged at 2500× *g* for 35 min and the supernatant was collected. The supernatant was treated in two different ways to formulate crude and purified rPA. The crude rPA (CrPA) supernatant was treated with Limulus Amoebocyte Lysate (LAL) endosafe endotoxin quantification and removal kit (ThermoFisher Scientific Rockford, IL, USA). To prepare PrPA, the supernatant was purified using Ni^2+^-TED column (Machery-Nagel, Düren, Germany) as directed by the manufacturer, followed by filtration using an LAL endosafe endotoxin Quantification and removal kit (ThermoFisher Scientific). The rPA protein was confirmed by SDS PAGE and Western blot using 4–20% protein gel (ThermoFisher Scientific), and the rPA protein yield was quantified with Pierce BCA protein assay kit (ThermoFisher Scientific) following the manufacturer’s protocol. The purified protein procedure was followed for rPA83 used in the ELISA.

To determine the amount of PA in the crude vaccine, 1 mL of CrPA was purified with the Ni^2^-TED column and protein concentration was determined using the Pierce BCA protein assay kit (ThermoFisher Scientific) according to the manufacture’s instruction. The results were used as a standard to calculate the concentration CrPA (Supplementary Material 1). The PrPA and CrPA concentrations were used in the vaccine formulation (see the section, “formulation of non-living anthrax vaccine”, below).

### 4.2. Formalin-Inactivated Spores (FIS) Preparation

*Bacillus anthracis* 34F2 spores from Onderstepoort Biological Products (OBP), South Africa, batch 863, was cultured as described by [16] and used to produce FIS for the vaccine. The spores were resuspended in PBS/0.1 g gelatin and stored at −80 °C. Aliquots of the FIS suspension were tested for sterility by plating-on blood agar after treatment with histidine to neutralize any remnant formalin. The spores used for in vivo mouse challenge were not inactivated with formalin.

### 4.3. Formulation of Non-Living Anthrax Vaccine

The formulation was produced in a large volume of 60 mL (60 doses) each for the crude and purified vaccine formulations. The antigens and PBS solution were ascetically pooled and mixed at 250 rpm with a magnetic stirrer, while 50% adjuvant volume (alhydrogel^®^) was slowly added and stirred for 2 h at room temperature, followed by slowly adding the remaining 50% adjuvant volume (emulsigen-D^®^) which was stirred for 2 h at room temperature. The vaccine formulation was transferred into sterile HDPE vaccine vials (10 mL) and retention vials were sent for a quality control test at Design Biologix. 

For the vaccine formulation, the rPA for both crude and purified concentration was determined with Pierce BCA protein assay kit (ThermoFisher Scientific) and the concentration of rPA for PrPA and CrPA vaccine formulations were calculated according to Supplementary Material 1. Then, it was adjusted through the FIS (10^8^ spores) and adjuvants to a volume of 1 mL. The aseptic vaccine formulation procedure was carried out according to the requirements for vaccines [48]. Briefly, the PrPA+FIS+adjuvants vaccine formulation constituted of 258 μL of 290 μg/mL PrPA (75 µg rPA), 400 μL of 2.5 × 10^8^ spores suspension per mL (1 × 10^8^ spores), 330 μL of adjuvants (Emulsigen-D^®^/Alhydrogel^®^ 1:1) and 12 µL PBS in the 1 mL dose recommended [48], whereas the CrPA+FIS+adjuvants vaccine formulation constituted of 517 μL of 146 μg/mL of rPA CrPA (75 µg rPA), 400 μL 2.5 × 10^8^ spore suspension (1 × 10^8^ spores), 330 μL of adjuvants (Emulsigen-D^®^/Alhydrogel^®^ 1:1) and 3 μL PBS. 

### 4.4. Immunization Animal Experiment and Passive Mouse Protection Tests

Cattle were screened for PA-reactive antibodies using the PA-ELISA. The cattle experiment was conducted on a farm where the animals were born and raised, as approved by the Director of Animal Health, South Africa under the biosecurity section 20 of the animal disease Act 35 of 1984 (registration number: 12/11/1/1/6). After subcutaneous treatment with 4 mL Ivermectin (Ivomec injection South African Reg. No. G1142 (Act 36/1947)) and intramuscular injection with 10 mL multivitamins (Kyroligo Reg No. G3087 (Act 36/174)), the cattle were randomly allocated to four different vaccination groups, with eight animals in each group, except for the negative control group consisting of four animals, according to the animal ethics approval (protocol number; V118-17 Amendment 1) (Table 2). The animals were fed ad libitum and examined daily by a veterinarian. One animal died (vaccine group (C4)) after serum was collected for week 12 during the experiment. The cause of death was diarrhoea caused by *Escherichia coli* infection, as revealed by the post-mortem examination and laboratory results.

The passive mouse protection test was conducted in the Onderstepoort Veterinary animal research unit (OVARU) facility, University of Pretoria, South Africa in accordance with ethical principles and guidelines provided by the animal ethical committee of the University of Pretoria (protocol number; V118-17, Amendment 1) and section 20, Act 35 of 1984 permission granted by the Directorate of Animal Health of South Africa (registration number; 12/11/1/1/6(909). The passive protection test challenge model consisted of naïve inbred A/J mice from Jackson Laboratory, Bar Harbor, ME, USA. The A/J mouse strain lacks the *Hc* gene encoding for complement component 5 (C5), which renders it vulnerable to systemic infection with the *B. anthracis* 34F2 Sterne vaccine strain spores [16]. The experimental design consisting of five mice per vaccinated cattle serum and three mice per negative control cattle is shown in Table 2. For the passive protection test, IgG was purified from sera collected from the vaccine groups at week 5, using the protein G spin column according to manufacturer’s instruction (NAb^TM^ Protein G Spin Kit, Thermo Scientific). The presence of anti-rPA specific IgG was confirmed using ELISA and the concentration of the IgG was measured with Pierce BCA protein assay kit (ThermoFisher Scientific). Purified IgG (500 µg) [49] was injected intraperitoneally into naïve A/J mice. The lethal challenge consisted of 2.16 × 10^5^ *B. anthracis* 34F2 strain spores in 200 μL injected through subcutaneous route, 24 h after the transfer of bovine polyclonal IgG. The A/J mice were monitored for clinical signs of anthrax intoxication for 14 days. Bacilli Giemsa stained smears and *B. anthracis* cultures from non-surviving A/J mice kidney, liver, and spleen on sheep blood agar were used to confirm death due to anthrax. Surviving mice were euthanized after 14 days by isoflurane overdose. 

### 4.5. Serum Immunoglobulin Titre Determination

Anti-rPA and anti-FIS antibodies (IgM, IgG, IgG1 and IgG2) sera titre from vaccinated cattle were determined by ELISA as previously described by Ndumnego et al. [16]. Briefly, 96-wells microtitre plates (Nunc immunoplate Maxisorp) were coated with 0.5 μg of rPA or 10^8^ FIS in carbonate-biocarbonate buffer (Sigma-Aldrich, USA) and incubated overnight at 4 °C. Plates were washed twice with wash buffer (PBS + 0.05% Tween 20 (PBST)) with ELISA microplate washer (Biorad PW40 France) and blocked with blocking buffer (PBST + 5% skimmed milk powder (PBSTM)) and incubated for 1 h at room temperature. Plates were washed twice and sera were added to the plate in duplicates across the plate at the starting concentration for IgG 1:100 and 1:50 for IgM and the immunoglobulin subclasses (IgG1 and IgG2) using PBSTM as sample dilution buffer. The plate was incubated on a shaking incubator at 160 rpm for 30 min at room temperature and washed five times. Each plate was conjugated with the appropriate secondary antibody. For IgG, the secondary antibody (goat anti-bovine IgG (ThermoFisher Scientific)) was diluted at the concentration of 1:10,000 in PTSMP, whereas for IgM, IgG1 and IgG2, sheep anti-bovine IgM (ThermoFisher Scientific), and sheep anti-bovine IgG1 and IgG2 (ThermoFisher Scientific) were all diluted at 1:4000 in PTSMP, respectively. This was followed by 30 min incubation at room temperature in a shaking incubator (160 rpm). After washing five times, the plates were developed with 2,2′ azino bis (3 ethylbenzthiazoline-6-sulfonic acid) diammonium salt (Sigma-Aldrich, St Louis, MO, USA) and absorbance readings taken on Biotek Powerwave XS2 reader at 405 nm. The reciprocal of the nearest serum dilution above the cut-off optical density (mean optical density of negative control serum + 3 SD) was considered as the endpoint titre of individual serum. Titres of <50 for IgM, IgG1, IgG2 and IgG were ascribed an arbitrary value of 10. The positive control was obtained from hyperimmune Sterne vaccinated animals that survived a virulent *B. anthracis* spores challenge [11] and the negative control is the pool of serum from pre-vaccination screened sera. 

### 4.6. Toxin Neutralization Assay (TNA)

The functional ability of antibodies from vaccinated cattle and controls to neutralize anthrax lethal toxin was assessed using an in vitro TNA, as previously described by Ndumnego et al. [16]. The TNA was determined with J774A.1 mouse macrophage cells (ECACC cat no 91051511) exposed to anthrax lethal toxin in the presence of antibodies. Briefly, 1.0 × 10^5^ J774A.1 macrophages per well in Dulbecco’s Modified Eagle Media (DMEM) and 10% foetal bovine serum (FBS) were incubated at 37 °C and 5% CO_2_ overnight in 96-well cell culture plates. Sera from each animal were 2-fold serially diluted in duplicates with the starting dilution of 1:50 in DMEM formulated with 5% FBS containing PA (500 ng/mL) and LF (400 ng/mL) (List Biological Laboratories Inc, Campbell, CA, USA) for 1 h at 37 °C and 5% CO_2_ before addition to the overnight-cultured confluent J774A.1 mouse macrophages and incubated for 3 h. Thereafter, 25 μL of 5 mg/mL MTT (3-(4,5 dimethylthiazol-2-yl)-2,5-diphenyltetrazolium bromide (Invitrogen) per well was added and incubated for 2 h at 37 °C and 5% CO_2_ in darkness. Acidified isopropanol (100 µl of 90% isopropyl alcohol, 0.5% SDS, 25 mM HCl) was added to each well of the plate, followed by pipetting up and down to dissolve the formazan crystals dye. Finally, the plates were rested for 5 min and the absorbance read at 540 nm with a Biotek power wave XS2 reader. The neutralization of each serum sample was calculated using the formula:

(1) $$NT_{50}=\frac{\left(sample-toxin\;control\right)}{\left(medium\;control-toxin\;control\right)\;}\times 100$$

The neutralization titres (NT_50_) were expressed as the reciprocal of the highest serum dilution at which the J774A.1 macrophage cells survival yielded 50% neutralization using the Gen5 data analysis software (Biotek Instruments). 

### 4.7. Opsonophagocytic Assay

The opsonophagocytic potential of induced antibodies was evaluated on RAW 264.7 macrophage cells as previously described with few modifications [50]. Briefly, heat-activated, refractile ungerminated *B. anthracis* spores (2.6 × 10^9^ spores/mL) were pre-incubated with 10-fold serial dilutions of the immune sera and sera from the negative control (NegCtl), for 30 min in 4 °C and then added to RAW 264.7 macrophage cells (5 × 10^5^ cells/well) and incubated for 45 min at 37 °C in 5% CO_2_. The macrophage cells were washed with sterile PBS (pH 7.4 ± 1) and incubated with DMEM containing 10% FBS and 10 μg/mL gentamicin at 37 °C in 5% CO_2_ for 30 min to remove vegetative bacilli. Subsequently, the macrophage cells were washed with sterile ice-cold PBS, incubated for 5 min in 100 μL 0.1% Triton® ×100 (ThermoFisher Scientific) to lyse the macrophages and plated on LB media to count viable cfu/mL. Data are presented as percentage spore uptake by the macrophage cells. Sera from pre-vaccination screening was used as the negative control, whereas the group vaccinated with SLSV from this study was used as the positive control.

### 4.8. Statistical Analysis

Gen 5 data analysis software (Biotek Instruments, Winooski, VT, USA) was used to generate 4-parametre logistic curves for the ELISA and TNA titres. The collected data were log-transformed using GraphPad prism version 8.3.0 software. The antibody titres between groups at different time points on ELISA and TNA were compared using unpaired Student *t*-test with a two-tailed *p*-value and Kruskal–Wallis test, followed by Dunn’s multiple comparisons test with adjusted *p*-value, was utilized for intra-group comparisons. The mean survival time of the challenged A/J mice was plotted using the Kaplan–Meier survival curve. A log-rank (Mantel-Cox) test was used to compare survival between different vaccination groups. All graphical elucidations and the analysis were done using GraphPad prism version 8.3.0 software.

## 5. Conclusions

Our study demonstrates the potential of NLAV (PrPA+FIS or CrPA+FIS) formulated with combination adjuvants (Emulsigen-D^®^/Alhydrogel^®^) to induce an immune response against *B. anthracis*’ toxins and spores in vaccinated cattle. The immune response by PrPA+FIS+Emulsigen-D^®^/Alhydrogel^®^ showed a significant level of protection in the passive mouse protection assay that is comparable to the SLSV. Because of the non-living feature of the vaccine, it is compatible with antibiotic treatment in the case of a disease outbreak. It can also be adopted for wildlife vaccination as well as in feedlots and is devoid of residual virulence in vaccinated animals. Finally, further study should compare the immunogenicity of NLAV with the current SLSV in field vaccination practice trials. 

## Figures and Tables

**Table 1:** The correlation between antibody titres in sera from vaccinated cattle and survival time to death of challenged mice after passive transfer of purified antibodies.

	Anti-rPA (PrPA+FIS)	Anti-rPA (CrPA+FIS)	Anti-rPA (SLSV)	Anti-FIS (PrPA+FIS)	Anti-FIS (CrPA+FIS)	Anti-FIS (SLSV)	TNA (PrPA+FIS)	TNA (CrPA+FIS)	TNA (SLSV)
**Pearson correlation**	0.8196 *	0.1237 ^ns^	0.9239 **	0.7883 *	0.3836 ^ns^	0.8518 **	0.8588 **	0.4564 ^ns^	0.8778 **
**Significance (2-tailed)**	0.0128	0.7704	0.0010	0.0201	0.3482	0.0073	0.0063	0.2556	0.0042

PrPA: Purified recombinant protective antigen, CrPA: Crude recombinant protective antigen, FIS: Formalin inactivated spores, NegCtl: Negative control vaccinated with Emulsigen-D^®^/Alhydrogel^®^ adjuvants. ** Correlation significant: 0.001 (2-tailed), * Correlation significant: 0.05 (2-tailed). ^ns^ Correlation not significant: 0.1234 (2-tailed).

**Table 2:** Animal trial vaccination, dosage and sampling schedules.

Vaccine Groups and Cattle Number (n)	Vaccine and Dose	Cattle Vaccination and Sampling Schedule				A/J Mice Used in Passive Mouse Challenge (n)
		Wk 0	Wk 3	Wk 5	Wk 12	
SLSV (n = 8)	SLSV vaccine with Anthravax^®^ (10^8^ spores)	±	±	+	+	5 mice/serum sample (n = 40)
PrPA+FIS (n = 8)	Purified rPA (75 µg) + FIS (10^8^ spores) + Emulsigen-D^®^/Alhydrogel^®^ adjuvants (33% v/v)	±	±	+	+	5 mice/serum sample (n = 40)
CrPA+FIS (n = 8)	Crude rPA (75 µg) + FIS (10^8^ spores) + Emulsigen-D^®^/Alhydrogel^®^ adjuvants (33% v/v)	±	±	+	+	5 mice/serum sample (n = 40)
NegCtl (n = 4)	Emulsigen-D^®^/Alhydrogel^®^ adjuvants/saline (33% v/v)	±	±	+	+	3 mice/serum sample (n = 12)

The two-step vaccine schedule of the cattle included vaccination at week 0 and week 3. ±; Blood collection before vaccination, +; Blood collection, SLSV; Sterne live spore vaccine, PrPA; Purified recombinant protective antigen, CrPA; Crude recombinant protective antigen, FIS; Formaldehyde inactivated spores, NegCtl: Negative control.

## Supplementary Materials

The following are available online at https://www.mdpi.com/2076-0817/9/7/557/s1, Supplementary material S1: Purified and crude rPA calculation for vaccine formulation.
