# Supplementary material for: Immunogenicity of Non-Living Anthrax Vaccine Candidates in Cattle and Protective Efficacy of Immune Sera in A/J Mouse Model Compared to the Sterne Live Spore Vaccine
Source: Pathogens. 2020 Jul 10;9(7):557. doi: 10.3390/pathogens9070557 (PMC7400155; doi:10.3390/pathogens9070557)
Supplement: Supplementary file 1 [file pathogens-09-00557-s001.pdf]

Supplementary Materials

Supplementary material 1: Purified and crude rPA calculation for vaccine formulation

The vaccine formulation for the purified and crude rPA was calculated using:

$$C1V1 = C2V2.$$

$C1$ = Primary concentration

$V1$ = Primary volume

$C2$ = Final concentration

$V2$ = Final concentration

The concentration of the CrPA used for the vaccine formulation was determined with the 1 ml volume of the supernatant of the lysed cells after discarding the pellets following centrifugation. The formulas used to determine the rPA concentration in the crude whole supernatant are:

$$tx\ concn = wl\ concn - PrPA\ concn$$

$$ubrPA\ concn = tx\ concn - ft\ concn$$

$$CrPA\ concn = ubrPA + PrPA\ concn$$

$tx\ concn$ = Total protein concentration without the purified rPA concentration

$wl\ concn$ = Whole protein concentration in supernatant after centrifugation

$PrPA\ concn$ = Purified rPA concentration after purification using Ni-TED column (Machery-Nagel, England)

$ubrPA\ concn$ = Concentration of unbind rPA concentration present in the flow through after passing the whole supernatant the Ni-TED column (Machery-Nagel, England).

$ft\ concn$ = Concentration of the all proteins present in the flow through Ni-TED column.

$CrPA\ concn$ = Concentration of rPA in the whole supernatant after centrifugation.

The CrPA vaccine was formulated as described for the PrPA vaccine formulation.
